# Paroxysmal sympathetic hyperactivity syndrome caused by *Streptococcus intermedius* meningoencephalitis in children: a case report and literature review

**DOI:** 10.3389/fped.2024.1480514

**Published:** 2024-12-11

**Authors:** Qiuling Huang, Ruoyi Zhou, Yean Zhang, Jie Li, Feng Yu

**Affiliations:** ^1^ Department of Pediatrics, Fifth School of Clinical Medicine of Zhejiang Chinese Medical University, Huzhou Central Hospital, Affiliated Central Hospital of Huzhou University, Huzhou, Zhejiang, China; ^2^ Department of Pharmacy, Fifth School of Clinical Medicine of Zhejiang Chinese Medical University, Huzhou Central Hospital, Affiliated Central Hospital of Huzhou University, Huzhou, Zhejiang, China

**Keywords:** paroxysmal sympathetic hyperactivity, children, streptococcus intermedius meningoencephalitis, diagnosis, case report

## Abstract

**Introduction and importance:**

Paroxysmal sympathetic hyperactivity (PSH) syndrome often occurs with severe traumatic brain injury. However, it can also occur during infections, such as severe bacterial meningoencephalitis in children. *Streptococcus intermedius* is an aggressive, virulent, opportunistic pathogen. This species can cause meningoencephalitis in children, as reported in a few cases.

**Case information:**

A five-year-old boy with no relevant past medical history was admitted to a hospital because of a fever and progressive disturbance of consciousness. His head computed tomography scan and magnetic resonance imaging revealed extensive brain damage and an intraventricular abscess. A next-generation sequencing technology test performed on his cerebrospinal fluid revealed that the child's meningoencephalitis was caused by *S. intermedius*. During treatment, the child had clinical manifestations such as fever, tachycardia, tachypnea, diaphoresis, and hypertension. Changes in muscle tone and abnormal posture, which were misdiagnosed as epilepsy at the early treatment stage, were also observed; however, anti-epileptic treatment was ineffective. The child was diagnosed with PSH and received the appropriate treatment, and his symptoms eventually improved.

**Conclusions:**

To our knowledge, this is the first case report on PSH induced by *S. intermedius* meningoencephalitis. Early identification, diagnosis, and treatment of PSH are crucial.

## 1 Introduction

Paroxysmal sympathetic hyperactivity (PSH) is a clinical syndrome characterized by hyperactive sympathetic nerve activity accompanied by posture or dystonia, and it mainly occurs in traumatic brain injury (TBI), anoxic brain injury, cerebrovascular disease, and other diseases. Having inadequate understanding, some patients do not receive timely and appropriate diagnosis and treatment (1). *Streptococcus intermedius* is a Gram-positive member of the β-hemolytic opportunistic bacterium family that causes infections ranging from mild (e.g., dental abscesses and sinusitis) to severe infections involving the head and neck, lungs, abdomen, and soft tissues. In children, *S. intermedius* often causes head and neck infections, and, in rare cases, meningitis (2). Herein, we present the case of a five-year-old child with PSH induced by *S. intermedius* meningoencephalitis misdiagnosed as epilepsy at the early stage.

## 2 Case presentation

**Figure 1:**
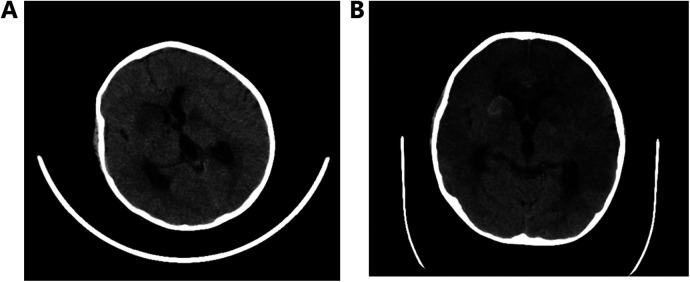
**(A)** Multiple low-density shadows in the right frontal lobe and bilateral basal ganglia and **(B)** hemorrhage in the right basal ganglia.

A five-year-old boy with no relevant past medical history was admitted to a hospital because of a fever and headache for 1 week and unconsciousness for 2 h. During physical examination, his body temperature, blood pressure, respiratory rate, pulse, and Glasgow Coma Scale score were 38.5°C, 105/60 mmHg, 30 beats per min, 108 per min, and 6 points, respectively. The boy also had neck stiffness (+), bilateral Pasteurelle sign (+), Krill's sign (+), increased limb muscle tone, and muscle strength (not cooperating). A computed tomography (CT) scan of the boy's head revealed multiple low-density shadows in the right frontal lobe and bilateral basal ganglia (Figure 1A). Hemorrhage soon appeared in the right basal ganglia (Figure 1B). The laboratory findings were as follows: white blood cell (WBC) count of 27.8 × 10^9 ^/L, NE% = 88%, and C-reactive protein (CRP) level of 67.4 mg/L. Cerebrospinal fluid (CSF) examination revealed bacterial infection, and next-generation sequencing showed that the patient tested positive for *S. intermedius*, as shown in Table 1. The final diagnosis was *S. intermedius* meningoencephalitis.

**Table 1:** Admission test results.

Test	Results	Reference range
Blood		
Leukocyte, 10^9 ^/L	27.8	4.4–11.9
NE,%	88.0	22–65
CRP, mg/L	67.4	<10
Blood culture	Negative	Negative
Cerebrospinal fluid		
Number of nucleated cells 10^6 ^/L,	3,800	<15
Multinucleate cell, %	90.0	–
Pan's test	Positive	Negative
Glucose mmol/L	1.61	2.70–4.20
Protein mg/L	630.9	120.0–400.0
Chloride ion mmol/L	115.3	107–137.0
Cerebrospinal fluid culture	Negative	Negative
Cerebrospinal fluid NGS	*Streptococcus intermedia*-positive	Negative

**Figure 2:**
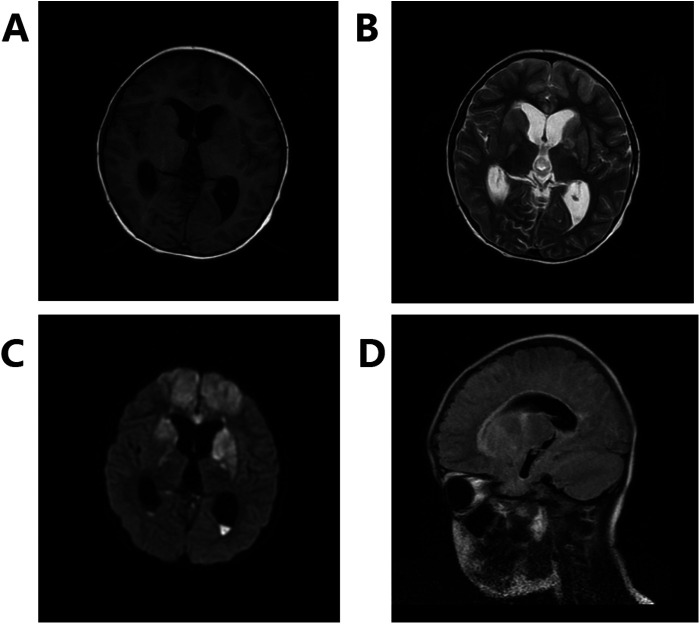
Diffuse cerebral hemispherical lesions, pus in the cisterna and ventricle, and midbrain aqueduct stenosis with supratentorial hydrocephalus. **(A)** Shows T1, **(B)** shows T2, **(C)** shows DWI, and **(D)** shows Flair.

After the identification of *S. intermedius* as the cause of the intermediate streptococcal infection, the patient was treated with vancomycin for two weeks. Subsequently, the patient's condition showed gradual improvement, with a decrease in fever, WBC count (reduced to 14.7 × 10^9 ^/L), CRP (reduced to 26.1 mg/L), and CSF cell count (decreased to 183 × 10^6 ^/L). However, despite these improvements, the patient continued to experience recurring fever and exhibited signs/symptoms, such as staring to the left, increased lower limb muscle tone, opisthotonic posture, tremors in the upper limbs, noticeable laryngeal phlegm, accelerated heart rate (up to 180 bpm), and frequent episodes lasting for several minutes multiple times a day. Although epileptic seizure was suspected, the 24 h continuous Video electroencephalogram (EEG) monitoring did not show any epileptic discharge. Treatment with midazolam, valproic acid, oxcarbazepine, and levetiracetam failed to control the seizures. Subsequent magnetic resonance imaging (MRI) revealed diffuse cerebral hemisphere lesions, pus in the cisterna and ventricle, midbrain aqueduct stenosis, and supratentorial hydrocephalus (Figure 2).

The patient underwent bilateral lateral ventricular irrigation and drainage following a referral to neurosurgery. After surgery, the child experienced intensified attacks characterized by fever (above 39°C), profuse sweating, a rapid heart rate of 180–200 bpm, elevated blood pressure of 180/100 mmHg, rapid breathing of 50–60 times per min, phlegm in the larynx, muscle stiffness, and opisthotonus. These attacks lasted 30–60 min, occurred dozens of times a day, and were often triggered or worsened by actions, such as suctioning phlegm, changing positions, patting on the back, and nasogastric feeding. Treatment with dexmedetomidine and propofol proved ineffective. After a multidisciplinary consultation involving neurosurgery, neurology, and pediatric neurology and based on Baguley's diagnostic criteria (1), the patient was determined to have a Clinical Feature Scale (CFS) score of 17 points, a Diagnosis Likelihood Tool (DLT) Scale score of 10 points, and a PSH Assessment Measure (PSH-AM) Scale score of 27. The final diagnosis was PSH.

**Figure 3:**
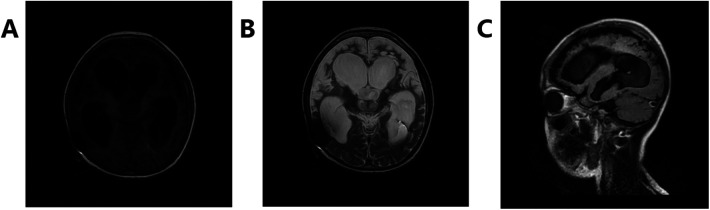
Changes in sequelae of encephalitis, bilateral cerebral hemisphere atrophy, partial softening, ventricle enlargement, and changes after drainage tube operation. **(A)** Shows T1, **(B)** shows T2, and **(C)** shows Flair.

Treatment included micropump-delivered midazolam and nasally administered propranolol, gabapentin, bromointine, and other medications. After surgery, linezolid was utilized to combat infection, resulting in a gradual decrease in body temperature to normal levels within a week and a reduction in the frequency of attacks. After six weeks of anti-infection therapy, a lateral ventriculoperitoneal shunt procedure was performed to address hydrocephalus. Subsequently, occasional attacks were managed with nightly oral clonazepam intake, along with hyperbaric oxygen therapy and rehabilitation training. A two-year follow-up MRI revealed bilateral cerebral hemisphere atrophy, partial softening, and ventricle enlargement post-encephalitis (Figure 3). The patient experienced residual motor and cognitive impairments, with no PSH recurrence.

## 3 Discussion

As an opportunistic pathogen, *S. intermedius* poses a high risk in individuals undergoing oral surgical procedures or those with sinusitis. The majority of infections are associated with abscess formation, with certain genotypes increasing the likelihood of spreading to the brain and causing brain abscesses (3). Reported cases also include brain abscesses (4) in children and meningitis (5). Treatment typically involves ceftriaxone and metronidazole, either alone or in combination with vancomycin (3). However, in some instances, aggressive anti-infective therapy may effectively halt disease progression. For localized abscesses, abscess drainage and surgery are considered the primary interventions, and surgical irrigation and drainage are utilized to achieve optimal infection control.

PSH is a severe clinical syndrome with an unknown pathogenesis that is characterized by mydriasis, shortness of breath, tachycardia, high fever, profuse sweating, increased blood pressure, and increased limb muscle tone (1). In adults, PSH is often secondary to central nervous system diseases, and children typically develop the condition as a result of brain injury. PSH has an overall prevalence of 13%–14% in children with acquired brain injury (6). PSH presents neither specific imaging characteristics nor laboratory indications. Various evaluation standards exist; however, the main diagnostic method is PSH-AM (consisting of CFS and DLT), which was proposed by Baguley et al. in 2014. When the total score of all symptoms (CFS + DLT) is greater than 17, a likely diagnosis is given (1). In the case presented in this study, the final score was 27 points, and the diagnosis was confirmed after ruling out symptoms caused by other conditions.

Therapeutic drugs primarily function by inhibiting hypersensitivity reactions in sensory pathways, reducing central sympathetic nerve outflow, and blocking peripheral organ responses to sympathetic nerves. Commonly used drugs include opioids, gamma-aminobutyric acid agonists, dopamine receptor agonists, beta-receptor antagonists, and benzodiazepines (7). For instance, propranolol acts as a β-receptor antagonist, leading to decreased blood pressure and heart rate. Bromocriptine, an agonist of dopamine receptors in the hypothalamus and pituitary gland, targets D2R, D3R, and D4R to induce a cooling effect (8). It is primarily utilized to address fever and dystonia in patients with PSH syndrome. Clonazepam, which does not cause sedation in small doses, can relax skeletal muscles, reduce muscle tension, lower the heart rate, ease respiratory muscles, and alleviate shortness of breath. Hence, it is used as a maintenance drug for late stages. Recent studies have shown that hydrogen sulfide levels in the paraventricular nucleus of the hypothalamus play a role in regulating sympathetic nerve efferent activity, thus offering a potential new strategy and target for PSH prevention and treatment (9).

In clinical practice, PSH is frequently misunderstood, which leads to potential missed or incorrect diagnoses. In this particular case, the child was initially misdiagnosed with epileptic seizures and did not respond to various antiepileptic medications, ultimately leading to prolonged hospitalization and increased medical costs.

## 4 Conclusion

PSH has been sporadically observed in adults with TBI; however, it rarely occurs in children. Herein, we present the first reported case of PSH induced by *S. intermedius* meningoencephalitis. PSH can be mistakenly diagnosed as epileptic seizures, malignant syndrome, or central hyperthermia at the initial stages (10). Hence, prompt identification and specific treatment are crucial to minimize hospitalization durations and medical costs.

## Data Availability

The raw data supporting the conclusions of this article will be made available by the authors, without undue reservation.
